# Low molecular weight fucoidan protects renal tubular cells from injury induced by albumin overload

**DOI:** 10.1038/srep31759

**Published:** 2016-08-22

**Authors:** Yingli Jia, Yi Sun, Lin Weng, Yingjie Li, Quanbin Zhang, Hong Zhou, Baoxue Yang

**Affiliations:** 1 State Key Laboratory of Natural and Biomimetic Drugs, Key Laboratory of Molecular Cardiovascular Sciences, Ministry of Education, and Department of Pharmacology, School of Basic Medical Sciences, Peking University, 100191, P.R. China; 2 Key Laboratory of Experimental Marine Biology, Institute of Oceanology, Chinese Academy of Sciences, Qingdao 266071, P.R. China

## Abstract

Albuminuria is a causative and aggravating factor for progressive renal damage in chronic kidney disease (CKD). The aim of this study was to determine if low molecular weight fucoidan (LMWF) could protect renal function and tubular cells from albumin overload caused injury. Treatment with 10 mg/g bovine serum albumin caused renal dysfunction, morphological changes, and overexpression of inflammation and fibrosis associated proteins in 129S2/Sv mice. LMWF (100 mg/kg) protected against kidney injury and renal dysfunction with decreased blood creatinine by 34% and urea nitrogen by 25%, increased creatinine clearance by 48%, and decreased significantly urinary albumin concentration. *In vitro* proximal tubule epithelial cell (NRK-52E) model showed that LMWF dose-dependently inhibited overexpression of proinflammatory and profibrotic factors, oxidative stress and apoptosis caused by albumin overload. These experimental results indicate that LMWF protects against albumin overload caused renal injury by inhibiting inflammation, fibrosis, oxidative stress and apoptosis, which suggests that LMWF could be a promising candidate drug for preventing CKD.

Chronic kidney disease (CKD), which is defined by glomerular filtration rate less than 60 mL/min/1.73 m^2^ or albuminuria more than 30 mg per day (or urinary albumin/creatinine ratio > 30 μg/mg), has become a public health issue. The global prevalence of CKD is 8~16%. CKD has been considered as an important independent risk factor for cardiovascular diseases and greatly increases risk and mortality of cardiovascular diseases^1^. Therefore, it is necessary to develop novel drugs to prevent and treat CKD.

Proteinuria or albuminuria is a common feature of CKD and often associates with glomerular dysfunction, tubular lesion and interstitial injury. Albuminuria is not only a marker of renal injury, but also a causative or aggravating factor for progressive renal damage^2^. Normally, most of filtered albumin (about 70%) is reabsorbed by the proximal tubules. Excessive reuptake of albumin can cause tubulointerstitial inflammation, oxidative stress, fibrosis, and tubular cell injury and death by activating a series of signaling pathways.

In animal models, albumin overload activates protein kinase C (PKC), mitogen-activated protein kinase (MAPK) and nuclear factor kappa B (NF-κB) pathways that lead to inflammation by increasing expression of fractalkine, monocyte chemotactic protein 1 (MCP-1) and RANTES (regulated upon activation, normal T-cell expressed and secreted)^3^,^4^,^5^,^6^. Inflammation and oxidative stress are key mediators in CKD^7^. Inflammation sets up the fibrotic stages through recruitment of inflammatory cells and activation of profibrotic factors. It has been found that albumin overload can induce overexpression of transforming growth factor-β1 (TGF-β1) and connective tissue growth factor (CTGF) and accumulation of extracellular matrix in proximal tubule cells^8^,^9^. Albumin can increase NADPH oxidase activity and generation of reactive oxygen species (ROS) in proximal tubule cells^10^. Moreover, tubular cell apoptosis, a common feature of end-stage renal disease, occurs in albumin overload models^11^,^12^,^13^,^14^.

Fucoidan represents a family of L-fucose-enriched sulfated polysaccharides. Low molecular weight fucoidan (LMWF, <10 kD) is obtained by free radical, mineral acid, organic acid, and enzymatic hydrolysis of fucoidan^15^. LMWF has multiple biological activities including anti-coagulant, anti-cancer, anti-inflammation, and anti-oxidation^16^,^17^,^18^. Our previous studies found that LMWF protects kidney from renal ischemia-reperfusion injury via inhibiting MAPK pathway^19^. Basing on its multi-biological activities, we hypothesize that LMWF could protect against albumin overload caused renal dysfunction and damages.

In the present study, *in vivo* and *in vitro* experiments were performed to investigate the effect of LMWF on renal injury caused by albumin and related mechanisms. Our experimental results suggest that LMWF may protect kidney from albumin overload caused renal dysfunction, inflammation, oxidative stress, apoptosis and fibrosis via multiple signaling pathways.

## Results

### LMWF protected against renal injury in albumin overload mice

**Figure 1:**
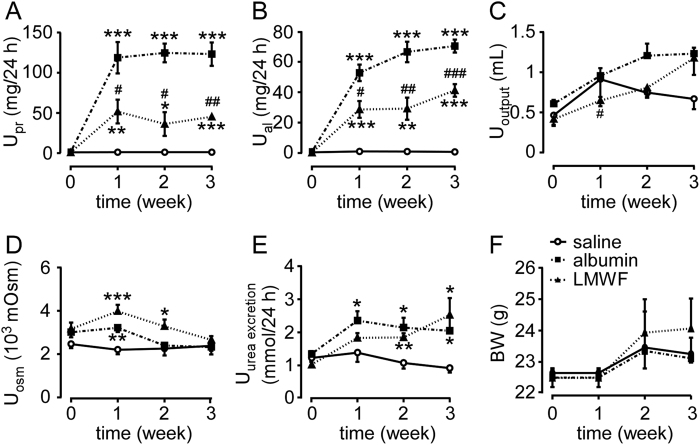
Urinary chemistry and body weight of experimental mice. 129S2/Sv mice were administrated with LMWF or saline twice daily for 7 days before bovine serum albumin (BSA) treatment. Albumin or saline was injected twice daily for 3 weeks. (**A**) Urinary protein (U_pr_). (**B**) Urinary albumin (U_al_). (**C**) Urine output (U_output_). (**D**) Urinary osmolality (U_osm_). (**E**) Urinary urea excretion (U_urea excretion_). (**F**) Body weight (BW). Means ± SEM, n = 5~6. *P < 0.05, **P < 0.01 and ***P < 0.001 *vs.* saline group. ^#^P < 0.05, ^##^P < 0.01 and ^###^P < 0.001 *vs.* albumin group (General linear model with repeated measures for A~F).

Renal injury model was established by daily intraperitoneal injections of bovine serum albumin (BSA) in 129S2/Sv mice. Urinary protein excretion (Fig. 1A), urinary albumin excretion (Fig. 1B), urine output (Fig. 1C), urinary osmalility (Fig. 1D) and urinary urea excretion (Fig. 1E) increased in albumin overload mice compared with control mice. The trend of urinary osmolality was contrary to that of urine output in each group, which indicates that urine concentrating ability was normal in albumin overload mice. The body weight of model mice and control mice was similar (Fig. 1F).

**Figure 2:**
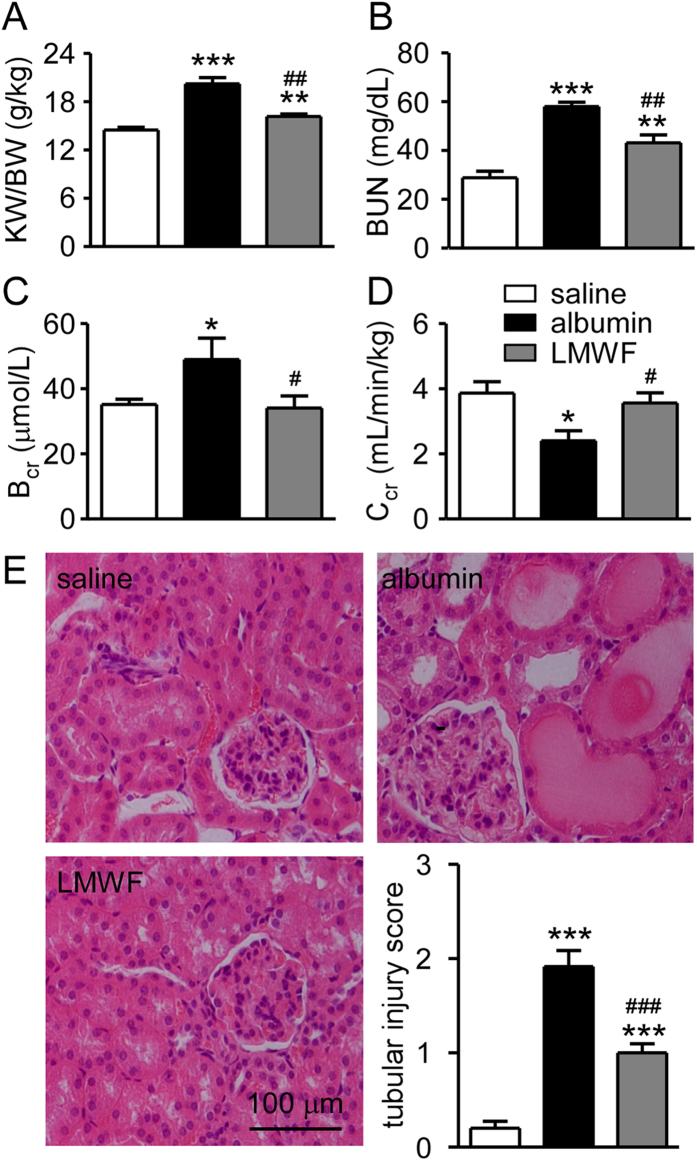
Blood chemistry, kidney index and renal morphology of experimental mice. Blood and kidney samples were collected and analyzed after albumin treatment for 3 weeks. (**A**) The ratio of kidney weight to body weight (KW/BW). (**B**) Blood urea nitrogen (BUN). (**C**) Blood creatinine (B_cr_). (**D**) Creatinine clearance (C_cr_). (**E**) Representative images stained with HE and tubular injury score. Values were means ± SEM. *P < 0.05, **P < 0.01 and ***P < 0.001 *vs.* saline group. ^#^P < 0.05, ^##^P < 0.01 and ^###^P < 0.001 *vs.* albumin group (ANOVA for A~E).

To determine the functional and structural changes in albumin overload mice, blood and kidney samples were collected after 3 weeks of daily albumin injections. The ratio of kidney weight to body weight (Fig. 2A), blood urea nitrogen and blood creatinine (Fig. 2B,C) were significantly increased and creatinine clearance (Fig. 2D) was significantly decreased in albumin overload group compared with control group. Figure 2E shows renal injury in tubules, including atrophy, flattening of epithelial cells and dilatation, and vacuolization in albumin treated group. The tubular injury score in the albumin overload group was elevated markedly compared with the control (Fig. 2E). All these results suggest that albumin overload causes renal functional and structural damages. The albumin overload mice are a proper experimental model for studying the protective effects of LMWF.

LMWF decreased significantly urinary protein and albumin levels (Fig. 1A,B). However, no significant difference was found in urine output (Fig. 1C), urinary osmalility (Fig. 1D), urinary urea excretion (Fig. 1E) and body weight (Fig. 1F) between the two groups. LMWF (100 mg/kg/day) significantly reduced kidney/body weight ratio by 20%, blood urea nitrogen by 25% and creatinine by 34%, and increased creatinine clearance by 48% in albumin overload mice (Fig. 2). LMWF also alleviated proximal tubule epithelial cell injury and decreased tubular injury score by 46% in albumin overload mice as shown in Fig. 2E. Besides, there was no significant difference in behavior, food and water consume among three groups. The data imply that LMWF can protect kidney tissue and renal function from albumin overload damage.

### LMWF reduced proinflammatory factors and fibrosis related proteins in albumin treated mice

**Figure 3:**
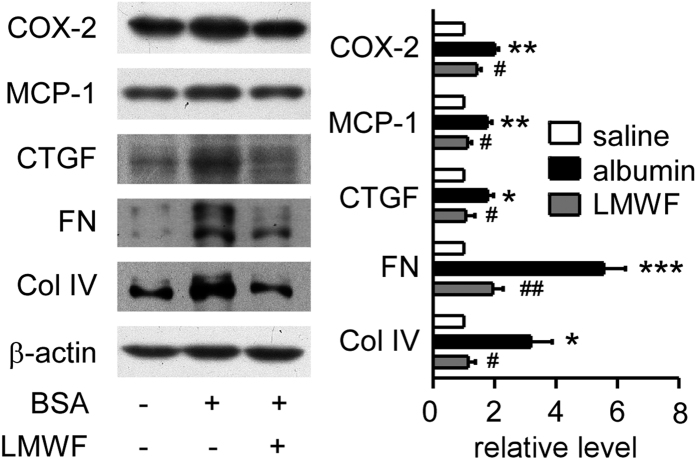
Effect of LMWF on expression of proinflammatory factors and fibrosis related proteins in mice. Representative blotting (left) and quantification of protein levels (right) are shown. Means ± SEM, n = 5~6. *P < 0.05, **P < 0.01 and ***P < 0.001 *vs.* saline group. ^#^P < 0.05, ^##^P < 0.01 *vs.* albumin group (ANOVA).

To test whether LMWF could influence inflammation and fibrosis in albumin overload mice, kidneys were collected after albumin treatment for 3 weeks and analyzed by Western blot. As shown in Fig. 3, expression levels of proinflammatory factors cyclooxygenase-2 (COX-2) and MCP-1 were significantly increased in albumin overload group. However, COX-2 and MCP-1 overexpression caused by albumin was suppressed by LMWF. Expression of CTGF, fibronectin (FN) and collagen IV (Col IV) was increased in albumin overload group as shown in Fig. 3. LMWF also reduced expression of these fibrosis-associated factors.

### LMWF reduced inflammation and fibrosis related proteins in albumin treated NRK-52E cells

**Figure 4:**
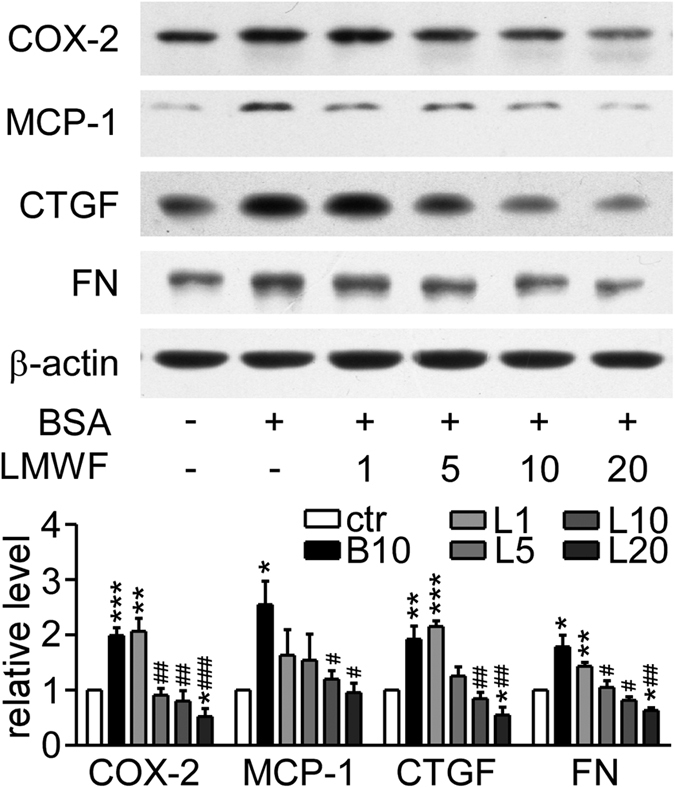
Effect of LMWF on inflammation and fibrosis related proteins in NRK-52E cells. Cells were incubated with 10 mg/mL albumin or PBS in the presence or absence of LMWF for 48 h and were collected for Western blot analysis. Representative blots (up) and relative protein levels (down) are shown. Means ± SEM, n = 3~4. *P < 0.05, **P < 0.01 and ***P < 0.001 *vs.* PBS control group. ^#^P < 0.05, ^##^P < 0.01 and ^###^P < 0.001 *vs.* albumin group (ANOVA).

NRK-52E cells were incubated with different concentrations of LMWF (1~20 μg/mL) under albumin (10 mg/mL) treatment for 48 h. COX-2 and MCP-1 significantly increased in cells treated with albumin (Fig. 4), which was consistent with *in vivo* data. LMWF concentration-dependently attenuated the albumin increased COX-2 and MCP-1 expression. LMWF also significantly reduced the overexpression of fibrosis-associated factors, CTGF and FN, in albumin treated cells in a dose-dependent manner.

### LMWF suppressed NF-κB pathway activation induced by albumin in NRK-52E cells

**Figure 5:**
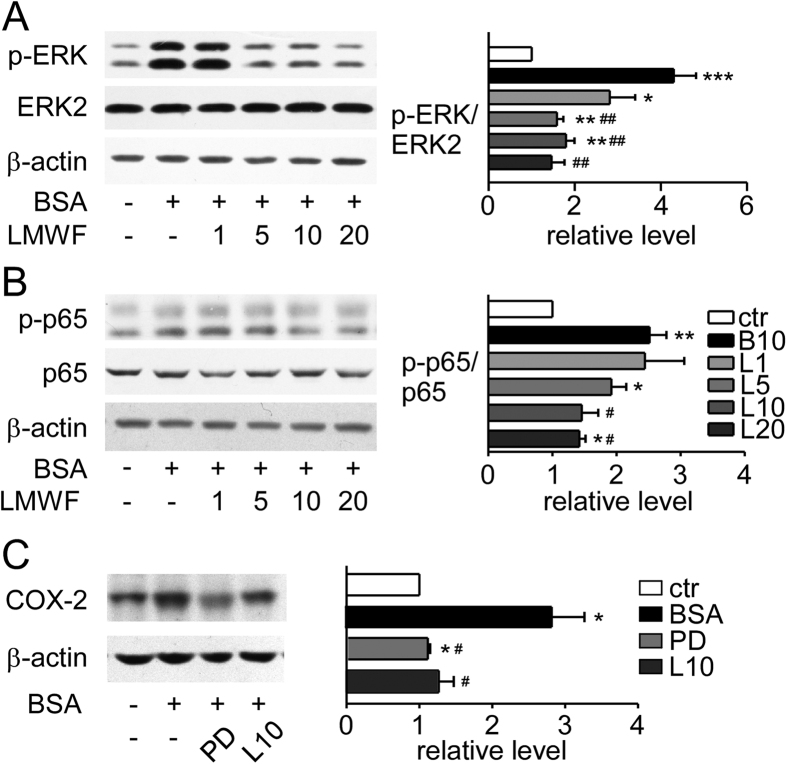
LMWF blocked NF-κB pathway activation induced by albumin in NRK-52E cells. (**A**) Expression levels of p-ERK, ERK2, and β-actin were detected by Western blot analysis. NRK-52E cells were pretreated with 1~20 μg/mL LMWF or PBS for 2 h and then were exposed to 10 mg/mL albumin or PBS for 15 min. Representative blots (left) and relative ratios of protein levels (right) are shown. (**B**) Expression levels of p-p65, p65, and β-actin in NRK-52E cells incubated with 1~20 μg /mL LMWF or PBS under 10 mg/mL albumin or PBS treatment for 48 h were determined by Western blot analysis. Left graph shows the representative blots and the right graph shows the density ratios. (**C**) Effect of ERK inhibitor PD98059 on albumin induced COX-2 expression. Cells were incubated with 10 mg/mL albumin or PBS in the presence or absence of LMWF or 20 μmol/L ERK inhibitor PD98059 (PD) for 48 h and were collected for Western analysis. Left graph shows the representative blots and the right graph shows the density ratios. Means ± SEM, n = 3~4. *P < 0.05, **P < 0.01 and ***P < 0.001 *vs.* PBS control group. ^#^P < 0.05 and ^##^P < 0.01 *vs.* albumin group (ANOVA).

Activated ERK could lead to NF-κB pathway activation that plays a central role in inflammation^3^. NRK-52E cells were pretreated with LMWF for 2 h and then incubated with albumin for 15 min. Western blot analysis showed that the expression levels of ERK2 did not change in each group. However, albumin increased significantly the phosphorylation of ERK that was significantly reduced dose-dependently by LMWF (Fig. 5A). As shown in Fig. 5B, LMWF inhibited significantly phosphorylation of p65 induced by albumin in a dose-dependent manner. To explore whether the activation of ERK signaling was involved in the increase of COX-2 expression, we detected the effect of ERK inhibitor PD98059 on albumin induced COX-2 expression. As shown in Fig. 5C, PD98059 at 20 μmol/L, as well as LMWF at 10 μg/mL, reversed the increased expression of COX-2 induced by albumin. The results suggest that COX-2 expression increased by albumin is partly dependent on the activation of ERK signaling pathway.

### LMWF suppressed oxidative stress caused by albumin in NRK-52E cells

**Figure 6:**
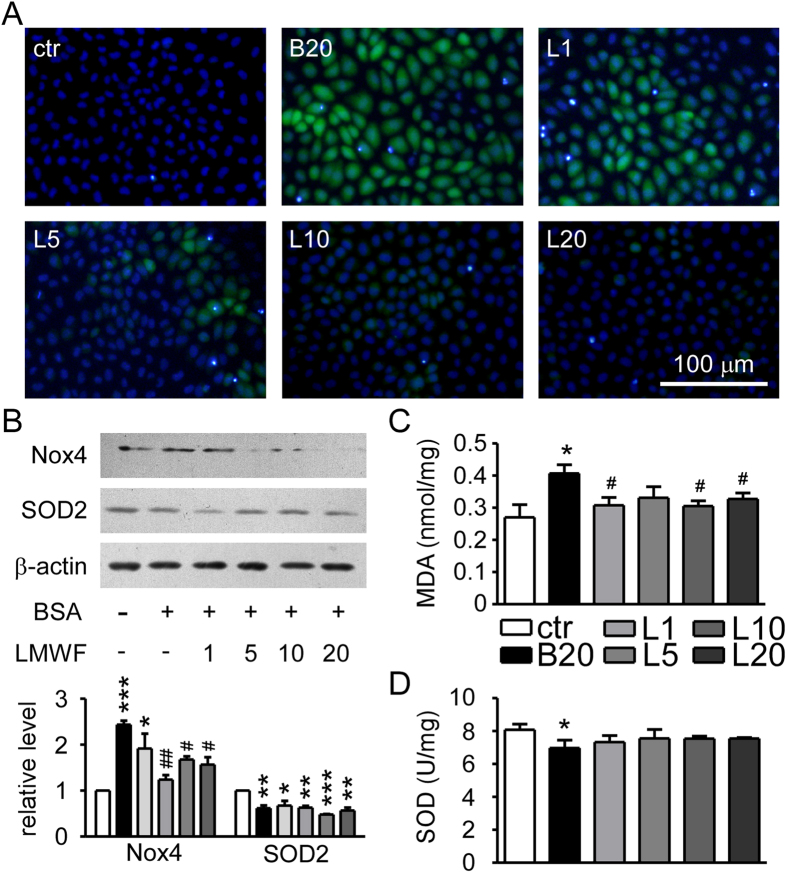
Effect of LMWF on oxidative stress induced by albumin in NRK-52E cells. (**A**) Formation of ROS. NRK-52E cells were incubated with LMWF under 20 mg/mL albumin treatment for 24 h. (**B**) Expression levels of Nox4, SOD2, and β-actin in NRK-52E cells incubated with 1~20 μg/mL LMWF under 20 mg/mL albumin for 24 h were determined by Western blot analysis. (**C**) MDA concentration in NRK-52E cells incubated with LMWF under 20 mg/mL albumin treatment for 72 h. (**D**) SOD activity in NRK-52E cells incubated with LMWF under 20 mg/mL albumin treatment for 72 h. Means ± SEM, n = 3~4. *P < 0.05, **P < 0.01 and ***P < 0.001 *vs.* PBS control group. ^#^P < 0.05, ^##^P < 0.01 *vs.* albumin group (ANOVA).

The effect of LMWF on oxidative stress induced by albumin overload was also determined in this study. Albumin (20 mg/mL) resulted in significantly elevated ROS level and NADPH oxidase 4 (Nox4) expression, which were decreased by LMWF (Fig. 6A,B). However, LMWF had no effect on decreased superoxide dismutase 2 (SOD2) expression induced by albumin (Fig. 6B). Increased MDA concentration and decreased SOD activity were found in albumin treated cells (Fig. 6C,D). LMWF reduced MDA concentration and had no effect on SOD activity.

### LMWF reduced albumin caused apoptosis in NRK-52E cells

**Figure 7:**
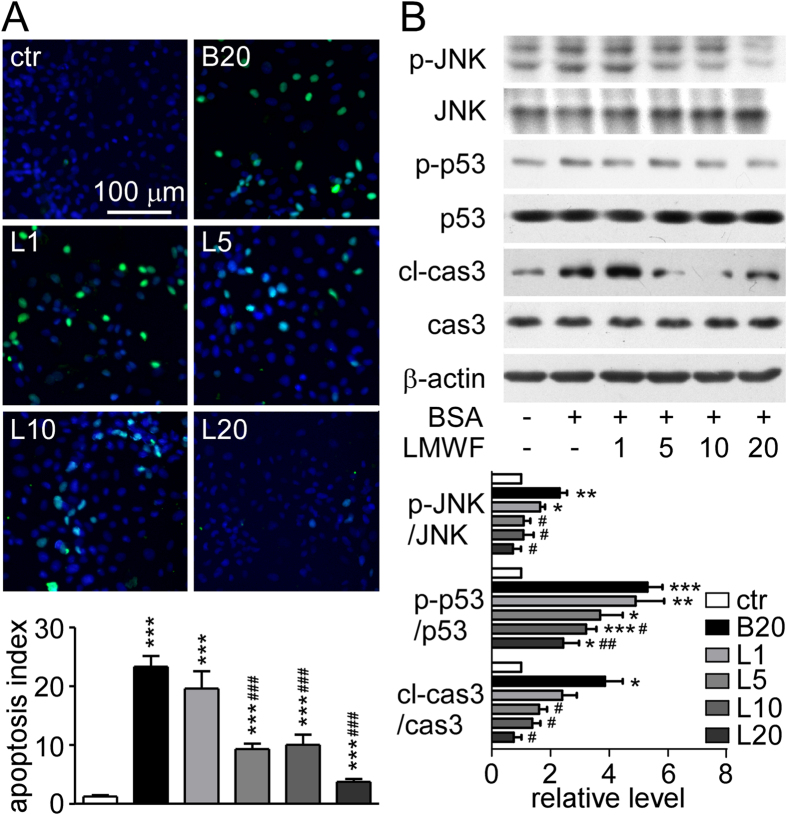
Effect of LMWF on apoptosis induced by albumin in NRK-52E cells. NRK-52E cells were incubated with LMWF under 20 mg/mL albumin treatment for 72 h. (**A**) Representative image (up) of TUNEL (green fluorescence) and Hoechst (Nuclei, blue fluorescence) staining of NRK-52E cells (original magnification ×400). The graph (down) shows the statistical analysis. (**B**) Representative blotting (up) and quantification (down) of apoptosis associated protein levels are shown. Means ± SEM, n = 3. *P < 0.05, **P < 0.01 and ***P < 0.001 *vs.* PBS control group. ^#^P < 0.05, ^##^P < 0.01 and ^###^P < 0.001 *vs.* albumin group (ANOVA).

The apoptotic NRK-52E cells were detected by TUNEL assay. TUNEL positive cells in albumin overload NRK-52E cells were more than solvent control group (Fig. 7A). LMWF reduced albumin caused TUNEL positive cells in a dose-dependent manner. Albumin significantly increased the expression levels of apoptosis related proteins, such as cleaved-caspase 3 (cl-cas3), and phosphorylation of JNK and p53, which were prevented by LMWF (Fig. 7B).

## Discussion

It is well known that proteinuria or albuminuria promotes the development and progression of CKD by inducing tubulointerstitial inflammation, oxidative stress, tubule cell injury and apoptosis, fibrosis and death^20^,^21^. Albumin overload in animals is often used as an experimental model to study functional and structural changes in CKD^22^,^23^. The present study confirmed that albumin overload caused renal dysfunction and structural damages in 129S2/Sv mice. In this study, we found that LMWF could effectively protect kidney from albumin overload induced functional and structural injuries.

The present study firstly focuses on the protective role of LMWF in inflammation induced by albumin. Clinical and experimental evidence demonstrated that inflammation accelerates CKD progression and involves activation of many genes. Among those, NF-κB is a critical transcription factor that can activate a large number of proinflammatory genes. It regulates the inflammatory process in CKD. Inhibition of NF-κB pathway by various agents, such as pyrrolidine dithiocarbamate^24^,^25^ and celastrol^26^, could lead to amelioration of renal injury, suggesting the importance of NF-κB as a therapeutic target of renal diseases. In this study, we found that LMWF could inhibit phosphorylation of NF-κB p65 subunit. Suppression of NF-κB activation subsequently down-regulates expression of COX-2 and MCP-1^27^. Our experimental results also show that LMWF could inhibit overexpression of MCP-1 and COX-2 in albumin overload mice and in NRK-52E cells treated with albumin. MCP-1, a potent chemoattractant for monocytes and macrophages to areas of inflammation, is able to influence inflammatory cells and fibroblasts leading to tubulointerstitial inflammation and fibrosis. NF-κB is also an upstream regulator of inflammation and fibrosis^28^. As a result, LMWF could protect the kidney by reducing inflammation.

Our previous studies showed that LMWF can ameliorate diabetic nephropathy in GK rats and STZ-induced diabetic rats by inhibiting epithelial-mesenchymal transition and fibrotic process^29^. We found that LMWF significantly reduced fibrotic factors, such as CTGF, FN, and Col IV, which were increased by albumin overload.

Studies have demonstrated that LMWF has a great potential as an antioxidant *in vivo* and *in vitro*. LMWF could reduce MDA content and increase SOD activity in diabetic GK rats and acute renal ischemia-reperfusion injury^19^,^30^. Besides, it also directly inhibits ROS production induced by high glucose in cultured rat cardiomyocytes^30^. As far as we know, oxidative stress is a contributor to the progression and development of CKD. For containing large numbers of mitochondria, renal proximal tubular epithelial cells are susceptible to ROS^31^. In pathologic conditions, a surplus of ROS in tissue leads to oxidative stress with various injurious consequences such as inflammation and fibrosis^32^. Nox4 expressed abundantly in renal proximal tubule^33^,^34^. MDA is a prominent product of lipid peroxidation and also a biomarker for oxidative stress^35^. We found that LMWF might inhibit albumin-induced oxidative stress through suppressing generation of ROS, MDA, and expression of Nox4.

It is demonstrated that apoptosis is induced by albumin in glomeruli and tubulointerstitium in Wistar rats after uninephrectomy^36^. Albumin induces apoptosis mainly through the extrinsic pathway, the intrinsic pathway and the endoplasmic reticulum stress pathway^11^,^12^,^14^,^37^. It was reported that 30 TUNEL-positive glomerular parietal epithelial cells of 241 glomeruli counted are found in Balb/c mice treated with 10 mg/g BSA for 3 weeks^38^. It implies that the number of apoptotic cells constitute a little proportion in mice treated with 10 mg/g albumin for 3 weeks. In our study, apoptosis was not found in albumin overload mice by measuring expression of apoptosis related proteins. However, albumin treatment significantly increased apoptosis in NRK-52E cells exhibiting elevated apoptotic index and increased apoptosis-associated proteins expression, which were reduced by LMWF treatment. Besides, LMWF also decreased the phosphorylation of JNK and p53, which suggests that LMWF might protect renal tubule cells from albumin induced apoptosis at least partly through the extrinsic pathway.

Fucoidan is a group of large molecules and has low bioavailability. LMWF, small pieces of fucoidan, exhibits more preferable biological activity. We demonstrate that LMWF has excellent potential to prevent renal injury in albumin overload CKD model for the first time. However, we did not find the key modulatory factors to link all pathogenic processes in renal injury caused by albumin. LMWF may act in several ways to protect against renal damage. *In vivo* use of LMWF was safe for mice due to no influence on body weight and general condition. Fucoidan does not show toxicity *in vitro* and *in vivo*^39^. It was reported that the dose of LMWF up to 2000 mg/kg body weight in mice is safe and has no significant genotoxic concern^40^.

In conclusion, our data indicate that LMWF may ameliorate albumin overload caused renal dysfunction and proximal cell damages mainly through inhibiting inflammation, fibrosis, oxidative stress and apoptosis. The experimental results suggest that LMWF may be a candidate drug for preventing and treating CKD.

## Materials and Methods

### Source of LMWF

LMWF was isolated from *L. japonica* commercially cultured in Qingdao, China, as described previously^41^. Its average molecular weight is about 7 kD determined by high performance steric exclusion chromatography analysis. LMWF (100 mg/mL) was dissolved in normal saline for animal treatment and in phosphate buffered saline (PBS) for cell incubation, and was filtered with 0.22 μm membrane filters.

### Albumin solution

For animal treatment, BSA (A7906, Sigma, St. Louis, MO) was dissolved in normal saline and the final concentration was 330 mg/mL. For cell incubation, albumin (200 mg/mL) solution was prepared in PBS. Both solutions were filtered with 0.22 μm membrane filters. Albumin solution was detected by using Limulus reagent kit (Xmhsjc, China) and had little endotoxin.

### Cell culture

Rat renal proximal tubule epithelial (NRK-52E) cells were purchased from the Cell Resource Center of Shanghai Institutes for Biological Sciences, Chinese Academy of Sciences. Cells were cultured in DMEM supplemented with 10% fetal bovine serum (GIBCO, Grand Island, NY), 100 U/mL penicillin, 100 μg/mL streptomycin in an atmosphere of 5% CO_2_/95% O_2_ at 37 °C. At 70~80% confluence, the cells were changed to serum-free medium and incubated for additional 24 h. The cells were treated with different concentrations of albumin, LMWF or ERK inhibitor PD98059 (513000, Calbiochem) for various periods of time as scheduled.

### TUNEL assay

NRK-52E cells were plated in 96-well culture plates. At 70~80% confluence, cells were incubated with 20 mg/mL albumin with or without LMWF at different concentrations (1, 5, 10, or 20 μg/mL) for 72 h. TUNEL assay was conducted using the *in situ* Cell Death Detection kit (Roche Applied Science) following the manufacturer’s instruction. The images were captured by Leica fluorescence microscope (Germany). Positive staining in ten sections per well from three wells was quantified. The apoptotic index was defined as (number of apoptotic cells/total number of nucleated cells × 100).

### Measurement of ROS

The formation of ROS was measured using DCFH-DA (2′, 7′-dichlorofluorescin diacetate) (D6883, Sigma, St. Louis, MO). NRK-52E cells were plated in 6-well dishes. At 70~80% confluence, cells were incubated with 20 mg/mL albumin with or without LMWF at different concentrations (1, 5, 10, or 20 μg/mL) for 24 h. Then the cells were treated with DMEM containing 20 μmol/L DCFH-DA. After incubation of 30 min at 37 °C, formation of ROS was detected. The images were captured by Leica fluorescence microscope (Germany).

### Animal model

Male 129S2/Sv mice weighting 21~24 grams, at age of 9 weeks, were acquired from the Animal Center of Peking University Health Science Center. The mice were maintained on a standard diet and had free access to water. For experiment, mice were housed in cages in a light, temperature and humidity controlled environment. We followed the established protocol for the albumin overload model in mice^22^,^42^. The mice were randomly divided into three groups: control group, albumin treated group, albumin and LMWF treated group. For albumin treatment, the mice were injected with normal saline for 1 week before albumin treatment and received injection of low endotoxin albumin for the next three weeks. The dosage of albumin was 2 mg/g body weight on the first day and was increased gradually to 10 mg/g body weight, 5 days later. For LMWF treatment, the mice were intraperitoneally injected twice daily with 100 mg/kg/day LMWF for 1 week and then received administration of LMWF for the next three weeks, when mice also received intraperitoneal injection of albumin twice daily in the same manner as albumin group. For control group, the mice received intraperitoneal injection of normal saline. The mice were placed in metabolic cages adapted for one day and 24-hour urine was collected every week. The body weight was measured weekly. Data were collected from 5~6 animals every group. All animal experiments were conformed to the Guide for the Care and Use of Laboratory Animals published by the US National Institutes of Health (NIH Publication, eighth Edition, 2011) and was approved by the Peking University Health Science Center Animal Experimentation Ethics Committee (Laboratory animal use license No. XYSK (JING) 2011-0039, Laboratory animal production license No. SCXK (JING) 2011-0012).

### Urine and blood chemistry

Urine and blood urea concentration was measured using QuantiChrom Urea Assay kit (BioAssay Systems Q20). Urinary osmolality was measured using freezing point depression (Micro-osmometer, FISKER ASSOCIATES Q23). Creatinine, albumin and total protein concentration in urine or blood were measured with commercial kits (NJJC Bio) according to the manufacturer’s instructions.

### Western blot analysis

Tissues or cells were lysed in RIPA lysis buffer containing protease inhibitor cocktail (Roche). Total protein was measured by BCA (Pierce). The lysates were electrophoresed on polyacrylamide gels and electrotransferred to polyvinylidene difluoride membranes (Amersham Biosciences). After blocking, the membranes were incubated with antibodies against p-ERK, ERK2, CTGF, p-JNK, JNK, β-actin (Santa Cruz), cl-cas3, caspase-3 (cas3), p-NF-κB p65, NF-κB p65 (Cell Signaling Technology), p-p53, p53, MCP-1, Col IV, SOD2, Nox4 (Abcam), or COX-2 (Bioworld). Goat anti-rabbit IgG or goat anti-mouse IgG (Santa Cruz) were added and the blots were developed with ECL plus kit (Amersham Biosciences). Quantitation was performed by scanning and analyzing the intensity of the hybridization band.

### Renal histological assessment

The kidneys were fixed with 4% paraformaldehyde and then embedded in paraffin for staining with haematoxylin-eosin (HE). Renal tubular damages were assessed using a tubular damage score, as previously described^43^. In brief, tubular injury was scored in a blinded manner according to the percentage of damages including atrophy and flattening of proximal tubule epithelial cells, and tubular dilation: 0 = normal; 1 = <20%; 2 = 20 to 40%; 3 = 40 to 60%; 4 = 60 to 80%; and 5 = 80%. The images were captured by Leica fluorescence microscope. Ten random pictures per kidney section were quantified.

### Statistical analysis

Statistical analysis was performed using SPSS software. The data were expressed as means ± SEM, and each experiment was performed at least three times. Statistical analysis was performed using one-way ANOVA followed by post hoc Bonferroni test, or General linear model with repeated measures followed by post hoc Bonferroni test. A “P value” < 0.05 was considered statistically significant.

## Additional Information

**How to cite this article**: Jia, Y. *et al*. Low molecular weight fucoidan protects renal tubular cells from injury induced by albumin overload. *Sci. Rep.* **6**, 31759; doi: 10.1038/srep31759 (2016).
